# Prognostic Predictive Characteristics in Patients With Fibrosing Interstitial Lung Disease: A Retrospective Cohort Study

**DOI:** 10.3389/fphar.2022.924754

**Published:** 2022-07-01

**Authors:** Yuanying Wang, Ziyun Guo, Ruimin Ma, Jingwei Wang, Na Wu, Yali Fan, Qiao Ye

**Affiliations:** ^1^ Clinical Center for Interstitial Lung Diseases, Beijing Institute of Respiratory Medicine, Beijing Chaoyang Hospital, Capital Medical University, Beijing, China; ^2^ Department of Occupational Medicine and Toxicology, Beijing Chaoyang Hospital, Capital Medical University, Beijing, China

**Keywords:** progressive fibrosing interstitial lung disease, prognosis, lung function test, blood count values, anti-fibrotic treatment

## Abstract

**Background:** Limited data are available regarding the entire spectrum of interstitial lung disease with a progressive fibrosing feature. We investigated the prevalence and prognostic predictive characteristics in patients with PF-ILD.

**Methods:** This retrospective cohort study included patients with fibrosing ILD who were investigated between 1 January 2015 and 30 April 2021. We recorded clinical features and outcomes to identify the possible risk factors for fibrosing progression as well as mortality.

**Results:** Of the 579 patients with fibrosing ILD, 227 (39.21%) met the criteria for progression. Clubbing of fingers [odds ratio (OR) 1.52, 95% confidence interval (CI) 1.03 to 2.24, *p* = 0.035] and a high-resolution computed tomography (HRCT)-documented usual interstitial pneumonia (UIP)-like fibrotic pattern (OR 1.95, 95% CI 1.33 to 2.86, *p* = 0.001) were risk factors for fibrosis progression. The mortality was worse in patients with PF with hypoxemia [hazard ratio (HR) 2.08, 95% CI 1.31 to 3.32, *p* = 0.002], in those with baseline diffusion capacity of the lung for carbon monoxide (DLCO) % predicted <50% (HR 2.25, 95% CI 1.45 to 3.50, *p* < 0.001), or in those with UIP-like fibrotic pattern (HR 1.68, 95% CI 1.04 to 2.71, *p* < 0.001).

**Conclusion:** Clubbing of fingers and an HRCT-documented UIP-like fibrotic pattern were more likely to be associated with progressive fibrosing with varied prevalence based on the specific diagnosis. Among patients with progressive fibrosing, those with hypoxemia, lower baseline DLCO% predicted, or UIP-like fibrotic pattern showed poor mortality.

## Introduction

The disease course in fibrosing interstitial lung disease (ILD) is highly heterogeneous and difficult to predict. Some patients experience a stable course or slowly functional decline while other patients may experience a rapidly progressive change (Cottin et al., 2018). Progressive fibrosing interstitial lung disease (PF-ILD) is a term referring to a group of diseases characterized by a high-resolution computed tomography (HRCT)-documented increase in the extent of pulmonary fibrosis, a decline in lung function, worsening respiratory symptoms and quality of life, and a high risk of early mortality despite available treatments, with a clinical course similar to that of idiopathic pulmonary fibrosis (IPF) (Cottin et al., 2018). PF-ILDs include a heterogeneous group of disorders associated with multifactorial etiology, including IPF, connective tissue disease-associated ILD (CTD-ILD), fibrotic hypersensitivity pneumonitis (FHP), unclassifiable idiopathic interstitial pneumonia (uIIP), and idiopathic non-specific interstitial pneumonia (Cottin et al., 2019). Reportedly, PF-ILDs may develop in approximately 33.0% of patients with fibrosing ILDs other than IPF (Olson et al., 2018; Nasser et al., 2021a; Ruaro et al., 2021; Lee et al., 2022). The estimated incidence of PF-ILD ranged from 4.0 to 4.7/100,000 person-years and the estimated prevalence from 6.6 to 19.4/100,000 individuals (Nasser et al., 2021a; Nasser et al., 2021b).

Several clinical trials have encompassed patients with progressive fibrosing, with the eligibility criteria for these studies helping to guide a standardized diagnosis of PF-ILD (Wongkarnjana et al., 2020). Although no uniform criteria were established, guidelines recommended monitoring of fibrosis progression based on evaluation of multiple components, such as a decline in lung function, increase in chest imaging-documented fibrosis, symptomatic worsening, and composite measures of these variables (George et al., 2020; Wongkarnjana et al., 2020; Raghu et al., 2022).

In view of the clinical and underlying physiological similarities between IPF and other PF-ILDs, anti-fibrotic agents, such as nintedanib or pirfenidone, are considered to be effective against progressive fibrosis (Flaherty et al., 2019; Gibson et al., 2020). However, watchful observation until the onset of HRCT-documented lung function decline and extensive fibrosis delays initiation of early anti-fibrotic therapy, and patients invariably present with clinically significant and irreversible injury. Several studies have investigated the clinical features and likely predictors in patients at high risk of PF-ILD at baseline (Kolb and Vašáková, 2019). Certain conditions, such as FHP (which is not associated with any specific antigen) and increasing age predispose to PF-ILD (Sharma et al., 2021; Churg, 2022). Smoking status was a risk factor for rheumatoid arthritis-ILD (RA-ILD) or primary Sjögren syndrome associated with ILD (Johnson, 2017; Wang et al., 2018). Male sex and a high baseline modified Rodnan skin score were strong predictors of forced vital capacity (FVC) decline in patients with systemic sclerosis-ILD (SSc-ILD) (Hoffmann-Vold et al., 2021). Risk factors for mortality have also been identified. Regardless of a specific diagnosis of ILD, patients with an HRCT-documented usual interstitial pneumonia (UIP) pattern or extensive traction bronchiectasis, FVC decline, or older age showed the highest mortality risk (Walsh et al., 2018; Yang et al., 2022). In addition, peripheral blood monocyte counts have been investigated in IPF and other ILDs as a predictor of prognosis (Misharin et al., 2017; Barratt et al., 2021). Although studies have reported findings for individual types of PF-ILDs (for example, SSc-ILD, RA-ILD, other CTD-ILDs, and FHP), limited data are available regarding the PF-ILDs as a whole group (Khanna et al., 2020; Skibba et al., 2020; Orlandi et al., 2022). Early accurate diagnosis of most ILDs was difficult. Studying clinical courses for the heterogeneous group of fibrosing ILDs as a whole group was essential to identify patients with the most rapidly progressive feature or poor prognosis and initiate early anti-fibrotic treatment.

The aims of this study were: 1) to investigate the prevalence and clinical features of the PF-ILDs and 2) to explore the potential factors associated with progression or mortality.

## Materials and Methods

### Study Design and Participants

**FIGURE 1:**
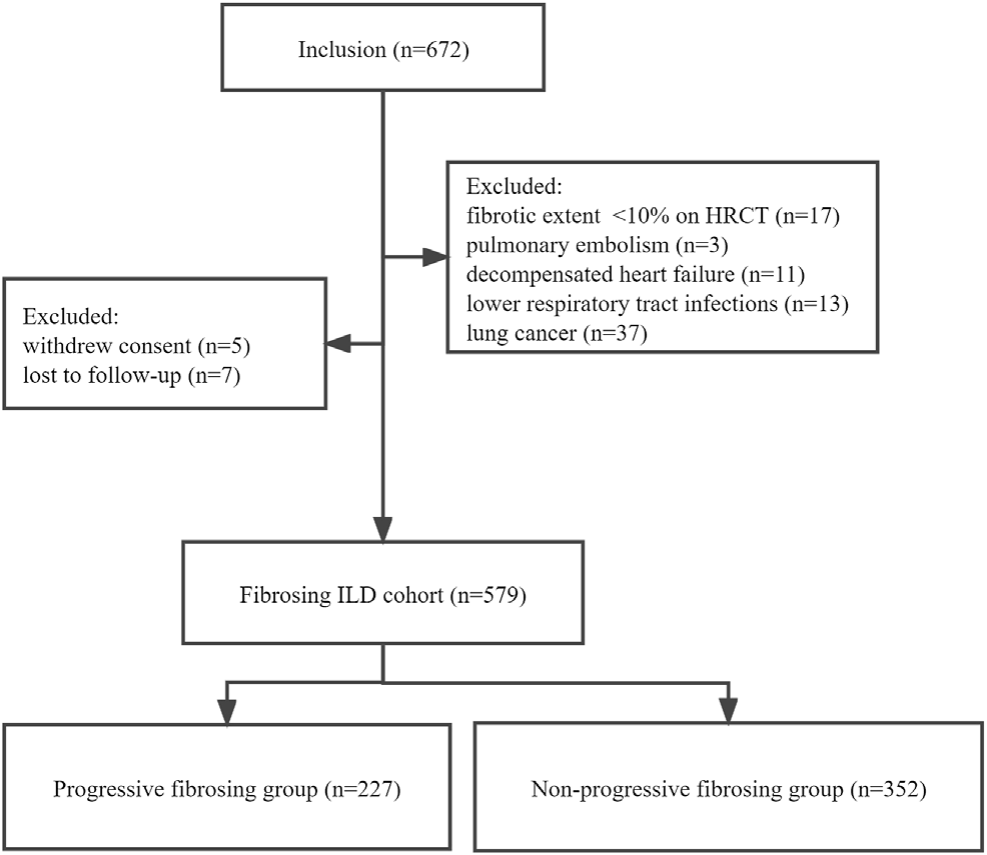
Patient flowchart. ILD, interstitial lung disease; PFT, pulmonary function test; HRCT, high-resolution computed tomography.

This observational retrospective cohort study was performed at Beijing Chao-Yang Hospital, China, a regional tertiary referral center specialized in the management of ILDs. We retrospectively screened all patients aged ≥18 years with a multidisciplinary diagnosis of fibrosing ILDs between 1 January 2015 and 30 April 2021. Multidisciplinary diagnoses were conducted between pulmonologists (QY, NW, and YW), radiologists (YF and JW), a rheumatologist (ZG), and a pathologist (RM) experienced in the diagnosis of ILD based on clinical characteristics, HRCT, and lung biopsy if appropriate. Patients whose results of baseline pulmonary function tests (PFTs) and HRCT were available were enrolled in the study. Following were the exclusion criteria: 1) <10% fibrosis documented on baseline HRCT, 2) diagnosis of pulmonary embolism, decompensated heart failure, or lower respiratory tract infections associated with disease progression, 3) lung cancer at baseline, 4) withdrawal of consent for study participation, and 5) loss to follow-up (Figure 1)*.* The study was registered at www.chictr.org.cn ChiCTR2100049247 and was approved by the Ethics Committee of Beijing Chao-Yang Hospital (2020-KY-437). All procedures were performed in accordance with the principles of the Declaration of Helsinki.

### Data Collection

We reviewed patients’ medical records to uniformly extract clinical data at the first clinical visit, including demographic information, physical examination, and routine clinical laboratory test results. Clinical and survival data were obtained from medical records, outpatient follow-up records (usually every 6–12 months), hospitalization details, and telephone interviews. Hypoxemia was defined as the partial pressure of oxygen in the arterial blood (PaO2) of less than 80 mm Hg obtained from the arterial blood gas (ABG) test at rest. The derivative blood cell count inflammation indexes included the neutrophil-to-lymphocyte ratio (NLR: neutrophils/lymphocytes), monocyte-to-lymphocyte ratio (MLR: monocytes/lymphocytes), platelet-to-lymphocyte ratio (PLR: platelet/lymphocyte), systemic inflammatory index (SII: neutrophils × platelets/lymphocytes), systemic inflammatory response index (SIRI: neutrophils × monocytes/lymphocytes), and aggregate index of systemic inflammation (AISI: neutrophils × platelets × monocytes/lymphocytes). The HRCT-documented UIP-like pattern included definitive UIP or a probable UIP pattern according to the Clinical Practice Guideline of IPF (Raghu et al., 2011; Raghu et al., 2018). Two thoracic radiologists (YF and JW) blinded to the clinical data independently determined whether the fibrosis involved >10% of the total lung and reviewed the HRCT scans by visual assessment. The disagreements were resolved *via* consensus. The kappa value for the interobserver correlation was 0.84.

The follow-up period ended on 30 April 2021. The primary outcome was progressive fibrosing. Patients who fulfilled any of the following criteria within 24 months despite administration of standard treatment were considered experiencing progressive fibrosis (Cottin et al., 2018; Flaherty et al., 2019): 1) a relative decline of ≥10% in FVC, 2) relative decline of ≥15% in diffusion capacity of the lung for carbon monoxide (DLCO), and 3) worsening symptoms and/or worsening radiological findings accompanied by a ≥5% to <10% relative decrease in FVC. The secondary outcome was all-cause mortality during the follow-up period. Survival was calculated from the time of the first visit to the outcome or the end of follow-up.

### Sample Size Calculation

PASS software, version 11.0 (NCSS, LLC. Kaysville, Utah, United States) was used to calculate the sample size. The sample allocation ratio was 1: 2 (PF: non-PF), given the estimated prevalence of PF-ILD among patients with fibrosing ILDs was 33.0% (Nasser et al., 2021a). We used a two-sided log-rank test for the 6-year all-cause mortality with 1-year accrual periods and an estimated 10.0% proportion of dropping out for both groups. An overall sample size of 260 patients (174 in the non-PF-ILD group and 86 in the PF-ILD group) was deemed sufficient to achieve >90% power at a 0.05% significance level to detect a hazard ratio (HR) of death with the 6-year mortality rate considered to be 40.0% for the PF group and 18.0% for the non-PF group (Adegunsoye et al., 2018). However, we attempted to enroll as many patients as possible within the study period. The final cohort included 579 patients, of which 227 patients were diagnosed with PF-ILD (85 deaths), fulfilling the 10-events-per-variable criterion of multivariable logistic regression and Cox analysis.

### Statistical Analysis

Statistical analyses were performed using SPSS Statistics software, version 26 (IBM, Inc., Chicago, IL, United States). Missing data were imputed using mean/median or removed for a complete case analysis. Data were expressed as means (SD) or medians (range), depending on the distribution. The Mann–Whitney U or *t*-test was used for continuous variables for comparison of intergroup differences and the chi-squared test or Fisher’s exact test for categorical variables. Linear mixed-effects analysis was applied to analyze disease behavior over time, with random terms for intercept and slope (for time-from-diagnosis). The covariance for the repeated measures was left autoregressive 1: heterogeneous, which yielded the best fit. Multicollinearity diagnostic tests were performed. Logistic regression analyses were used to investigate risk factors for the PF-ILD, and continuous variables were converted into dichotomous variables mainly through the median cut-off. The cut-off value was 70.0% for FVC% predicted and 50.0% for DLCO% predicted (Abe et al., 2021; Lacedonia et al., 2022). Survival curves were obtained using the Kaplan–Meier method, and Cox proportional analyses were performed to identify prognostic factors for mortality. Proportional hazards in the Cox analysis were checked using the Schoenfeld residual test. A *p*-value < 0.05 was considered statistically significant.

## Results

### Demographic and Clinical Characteristics

In this study, 277 (39.21%) out of 579 ILD patients met the criteria for PF-ILD; 64, 88, and 108 patients met criteria 1, 2, and 3, respectively; 28 patients met both the criteria 1 and 2, and 16 patients met both the criteria 2 and 3 (Supplementary Figure S1). Statistically significant baseline differences were observed in age (*p* = 0.001), underlying diagnosis (*p* < 0.001), clubbing of fingers (*p* = 0.005), and HRCT-documented UIP-like fibrotic pattern (*p* < 0.001) between the PF-ILD and non-PF-ILD groups (Table 1). Baseline blood count values did not differ significantly between patients with PF and non-PF. Within the PF-ILD group, gender, age, smoking status, UIP-like fibrotic pattern, FVC, GAP stage distribution, and anti-fibrotic treatment were statistically significant between IPF and non-IPF patients (Table 2). Comorbidity information for the whole cohort is shown in Supplementary Figure S2. The prevalence for pulmonary hypertension was 8.81% in the PF group and 5.11% in the non-PF group*.*

**TABLE 1:** Demographics and clinical characteristics of the cohort.

	All	PF-ILD group	Non-PF-ILD group	*p*-value
Number	579	227 (39.21)	352 (60.79)	
Male, *n* (%)	296 (51.12)	121 (53.30)	175 (49.72)	0.399
Age, years (IQR)	62 (55–68)	64 (57–70)	62 (54–68)	0.001
BMI, kg/m^2^ (SD)	25.90 (3.31)	25.92 (3.50)	25.89 (3.19)	0.907
Ever smoker, *n* (%)	233 (40.24)	98 (43.17)	135 (38.40)	0.248
Diagnosis, *n* (%)				<0.001
IPF	145 (25.04)	77 (33.92)	68 (19.32)	
CTD-ILD	297 (51.30)	109 (48.02)	188 (53.41)	
FHP	56 (9.67)	16 (7.05)	40 (11.36)	
uIIP	81 (13.99)	25 (11.01)	56 (15.91)	
Signs, *n* (%)				
Velcro crackles	531 (91.71)	214 (94.27)	317 (90.06)	0.072
Clubbing of fingers	154 (26.60)	75 (33.04)	79 (22.44)	0.005
Blood count values				
WBC (*n* * 10^9^/L)	7.60 (5.98–8.53)	7.40 (5.69–8.63)	7.77 (6.28–8.52)	0.132
Neutrophils (*n* * 10^9^/L)	4.87 (3.46–8.50)	4.54 (3.23–5.97)	5.06 (3.68–5.66)	0.156
Lymphocytes (*n* * 10^9^/L)	1.91 (1.42–2.30)	1.91 (1.35–2.29)	1.91 (1.46–2.30)	0.622
Monocyte (*n* * 10^9^/L)	0.47 (0.37–0.54)	0.47 (0.35–0.55)	0.48 (0.38–0.54)	0.293
RDW	13.20 (12.60–13.99)	13.20 (12.70–13.99)	13.20 (12.52–13.99)	0.570
LMR	3.90 (3.22–5.26)	3.90 (3.14–5.07)	3.90 (3.27–5.27)	0.393
NLR	2.68 (1.84–3.42)	2.60 (1.74–3.46)	2.68 (1.88–3.40)	0.914
PLR	121.68 (92.16–159.01)	121.68 (90.36–157.86)	121.68 (94.10–163.73)	0.214
SIRI	1.31 (0.76–1.65)	1.25 (0.73–1.85)	1.31 (0.77–1.55)	0.265
AISI	305.26 (145.40–396.00)	286.60 (139.56–433.36)	305.26 (156.84–377.23)	0.947
Pulmonary function (SD)				
FVC (L)	2.64 (0.80)	2.64 (0.79)	2.64 (0.82)	0.932
FVC% pred	85.45 (19.72)	85.94 (19.53)	85.15 (19.86)	0.633
DLCO% pred	60.26 (18.61)	58.94 (18.82)	61.11 (18.46)	0.171
UIP-like fibrotic pattern on HRCT, n (%)	163 (28.15)	86 (37.89)	77 (21.88)	<0.001
Hypoxemia, *n* (%)	119 (20.55)	54 (23.80)	65 (18.50)	0.122
Hospitalization at baseline, n (%)	162 (27.98)	70 (30.84)	92 (26.14)	0.219
Hospitalization frequency, n (IQR)	0 (0.1)	1 (0.1)	0 (0.1)	<0.001
Hospitalization frequency, *n* (%)				0.002
0	298 (51.47)	101 (44.49)	197 (55.97)	
1–2	236 (40.76)	99 (43.61)	137 (38.92)	
≥3	45 (7.77)	27 (11.89)	18 (5.11)	
Death, *n* (%)	128 (22.11)	85 (37.45)	43 (12.22)	<0.001
Median duration of follow-up, years (IQR)	3 (2.4)	3 (2.5.4)	3 (2.4)	0.054

Data were presented as mean ± SD or *n* (%) or median (IQR).

AISI, aggregate index of systemic inflammation; BMI, body mass index; CTD, connective tissue disease; DLCO, diffusion capacity of the lung for carbon monoxide; FHP, fibrotic hypersensitivity pneumonitis; FVC, forced vital capacity; HRCT, high-resolution computed tomography; iNSIP, idiopathic non-specific interstitial pneumonia; IPF, idiopathic pulmonary fibrosis; MLR, monocyte-to-lymphocyte ratio; NLR, neutrophil-to-lymphocyte ratio; PF, progressive fibrosing; PLR, platelet-to-lymphocyte ratio; RDW: red cell distribution width; SIRI, systemic inflammatory response index; uIIP, unclassifiable idiopathic interstitial pneumonia; UIP, usual interstitial pneumonia; WBC, white blood cell.

**TABLE 2:** Demographics and clinical characteristics of patients within the PF group.

	IPF	Non-IPF	*p*-value
Number	77	150	
Male, *n* (%)	60 (77.92)	61 (40.67)	<0.001
Age, years (IQR)	66 (60–74)	63 (55–68)	<0.001
BMI, kg/m^2^ (SD)	26.29 (3.19)	25.73 (3.65)	0.255
Ever smoker, *n* (%)	55 (71.43)	43 (28.67)	<0.001
Signs, *n* (%)			
Velcro crackles	29 (37.70)	46 (30.67)	0.337
Clubbing of fingers	71 (92.20)	143 (95.33)	0.289
Pulmonary function (SD)			
FVC (L)	2.80 (0.66)	2.45 (0.81)	0.001
FVC% pred	82.96 (16.86)	87.46 (20.67)	0.100
DLCO% pred	56.61 (19.47)	60.14 (18.43)	0.180
UIP-like fibrotic pattern on HRCT, n (%)	65 (84.42)	21 (14.00)^#^	<0.001
Hypoxemia, *n* (%)	19 (24.68)	35 (23.33)	0.822
GAP score	3 (1–2)	2 (1–3)	
Stage I (%)	55 (71.43)	123 (82.00)	
Stage II (%)	19 (24.68)	26 (17.33)	
Stage III (%)	3 (3.90)	1 (0.67)	0.077
Anti-fibrotic treatment (%)	42 (54.55)	31 (20.67)	<0.001

Data were presented as mean ± SD or *n* (%) or median (IQR).

#: 29 patients with CTD-ILD, 1 patient with FHP, and 1 patient with uIIP were included.

BMI, body mass index; DLCO, diffusion capacity of the lung for carbon monoxide; FVC, forced vital capacity; HRCT, high-resolution computed tomography; IPF, idiopathic pulmonary fibrosis; PF, progressive fibrosing; UIP, usual interstitial pneumonia.

### Prevalence of Progressive Fibrosing Interstitial Lung Disease

The most frequent diagnoses included CTD-ILD (*n* = 297%, 51.30%), IPF (*n* = 145%, 25.04%), uIIP (*n* = 81%, 13.99%), and FHP (*n* = 56%, 9.67%) (Table 1). The prevalence of PF-ILD in patients with ILDs, including those with IPF (*n* = 77), CTD-ILD (*n* = 109), FHP (*n* = 16), and uIIP (*n* = 25) was 53.10%, 36.70%, 28.57%, and 30.86%, respectively (Supplementary Figure S3).

### Treatments and Hospitalization

In the entire cohort, 74.96% of patients (*n* = 434) received at least one of the following treatments: administration of glucocorticoids, immunosuppressive agents, anti-fibrotic treatment, and oxygen therapy. Anti-fibrotic treatment was administered to 32.16% of patients with PF-ILD (*n* = 73) (Supplementary Table S1). In the PF group, 42 (54.50%) of IPF patients and 31 (20.70%) of non-IPF patients (29 CTD-ILD, 1 FHP, and 1 uIIP) received anti-fibrotic treatment (Table 2). Patients hospitalized within 1 month of the first visit were considered hospitalized at baseline. We observed that 298 (51.47%) patients underwent hospitalization at least once during follow-up, and 27 (11.89%) of 227 patients in the PF-ILD group vs. 18 (5.11%) of the 352 patients in the non-PF group underwent hospitalization at least thrice (Table 1).

### Lung Function Changes

**FIGURE 2:**
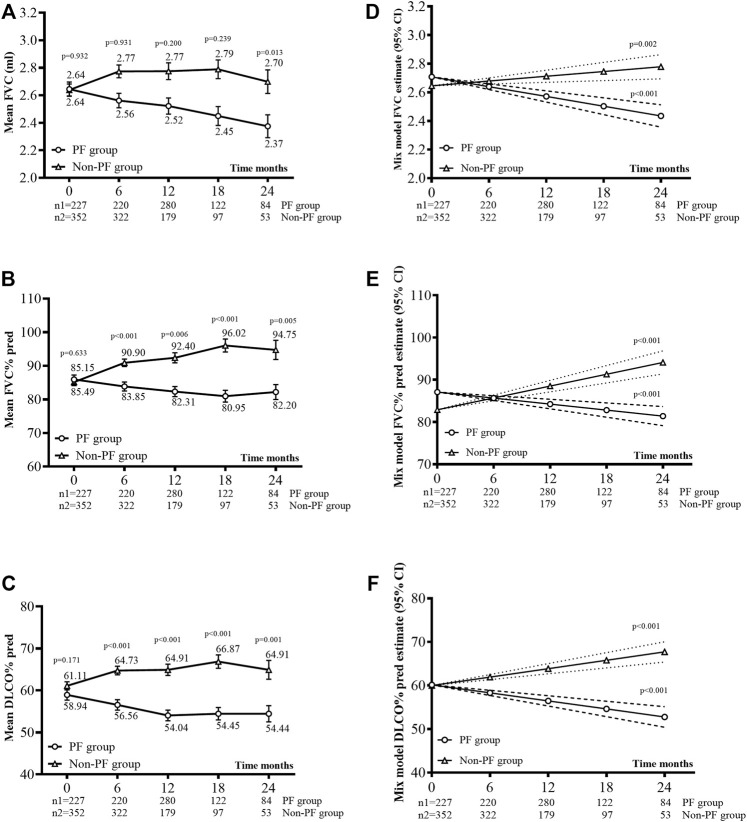
Lung function changes. **(A)**, **(B)**, and **(C)** Mean FVC ml (±SE), mean FVC% predicted (±SE), and mean DLCO% predicted (±SE) during observation. **(D)**, **(E)**, and **(F)** Disease behavior (FVC, FVC% pred, and DLCO% pred) over time was analyzed using a linear mixed model for each group, with a random effect for the intercept and slope. The covariance for the repeated measures was left AR (autoregressive)1: heterogeneous, as this yielded the best fit.

Figure 2 showed the mean lung function measures across different time intervals. The slope of FVC differed between the PF-ILD (−0.07 L/6 months, 95% CI −0.09 to −0.05) and non-PF-ILD (0.01 L/6 months, 95% CI 0.01–0.05) subgroups (*p* = 0.001) (Figure 2D). The slope of FVC% predicted in the PF-ILD group was −1.42%/6 months (95% CI −1.99 to −0.86), whereas that in the non-PF-ILD group was 2.80%/6 months (95% CI 2.12–3.49) (*p* = 0.04) (Figure 2E). The slope of DLCO% predicted also differed between subgroups (−1.84%/6 months, 95% CI −2.43 to −1.25 in the PF-ILD group vs. 1.93%/6 months, 95% CI 1.35 to 2.51 in the non-PF-ILD group, *p* = 0.003) (Figure 2F).

### Predictive Characteristics Associated With Progressive Fibrosing

Table 3 shows the results of logistic regression analysis for predictors of progressive fibrosing. Multivariable analysis was performed on significant factors obtained from univariate analysis with a *p*-value < 0.2. Although the NLR (OR 0.69, 95% CI 0.55 to 1.08, *p* = 0.039) was significantly associated with PF, the OR included the value 1. Patients with clubbing of fingers (OR 1.52, 95% CI 1.03 to 2.24, *p* = 0.035) and an HRCT-documented UIP-like fibrotic pattern (OR 1.95, 95% CI 1.33 to 2.86, *p* = 0.001) showed a high risk of fibrosis progression.

**TABLE 3:** Factors associated with progressive fibrosing.

Covariate	Univariable analysis		Multivariable analysis	
	OR (95% CI)	*p-*value	OR (95% CI)	*p*-value
Age	1.43 (1.02–2.00)	0.039	1.21 (0.85–1.73)	0.294
Male	1.16 (0.83–1.61)	0.400	—	—
BMI	0.85 (0.60–1.20)	0.351	—	—
Smoking	1.22 (0.87–1.71)	0.249	—	—
FVC% pred	0.69 (0.46–1.06)	0.090	0.72 (0.46–1.11)	0.134
DLCO% pred	1.24 (0.87–1.76)	0.237	—	—
Velcro	1.15 (0.82–1.61)	0.406	—	—
Clubbing of fingers	1.71 (1.17–2.48)	0.005	1.52 (1.03–2.24)	0.035
Hypoxemia	1.38 (0.92–2.07)	0.123	1.38 (0.90–2.11)	0.135
Hospitalization at baseline	1.26 (0.87–1.82)	0.219	—	—
UIP-like pattern on HRCT	2.18 (1.51–3.15)	<0.001	1.95 (1.33–2.86)	0.001
WBC	0.82 (0.59–1.15)	0.249	—	—
Neutrophils	0.75 (0.53–1.04)	0.086	0.76 (0.54–1.08)	0.122
Lymphocytes	0.82 (0.59–1.14)	0.237	—	—
Monocyte	0.88 (0.63–1.23)	0.464	—	—
RDW	0.98 (0.70–1.37)	0.913	—	—
LMR	1.08 (0.77–1.51)	0.656	—	—
NLR	0.69 (0.49–0.96)	0.028	0.69 (0.55–1.08)	0.039
PLR	0.94 (0.67–1.32)	0.712	—	—
SIRI	0.85 (0.61–1.19)	0.354	—	—
AISI	1.03 (0.73–1.46)	0.856	—	—

AISI: aggregate index of systemic inflammation; BMI, body mass index; CTD, connective tissue disease; DLCO, diffusion capacity of the lung for carbon monoxide; FVC: forced vital capacity; HRCT, high-resolution computed tomography; MLR, monocyte-to-lymphocyte ratio; NLR, neutrophil-to-lymphocyte ratio; PLR, platelet-to-lymphocyte ratio; RDW: red cell distribution width; SIRI, systemic inflammatory response index; UIP, usual interstitial pneumonia; WBC, white blood cell.

### Predictive Characteristics Associated With Survival

**FIGURE 3:**
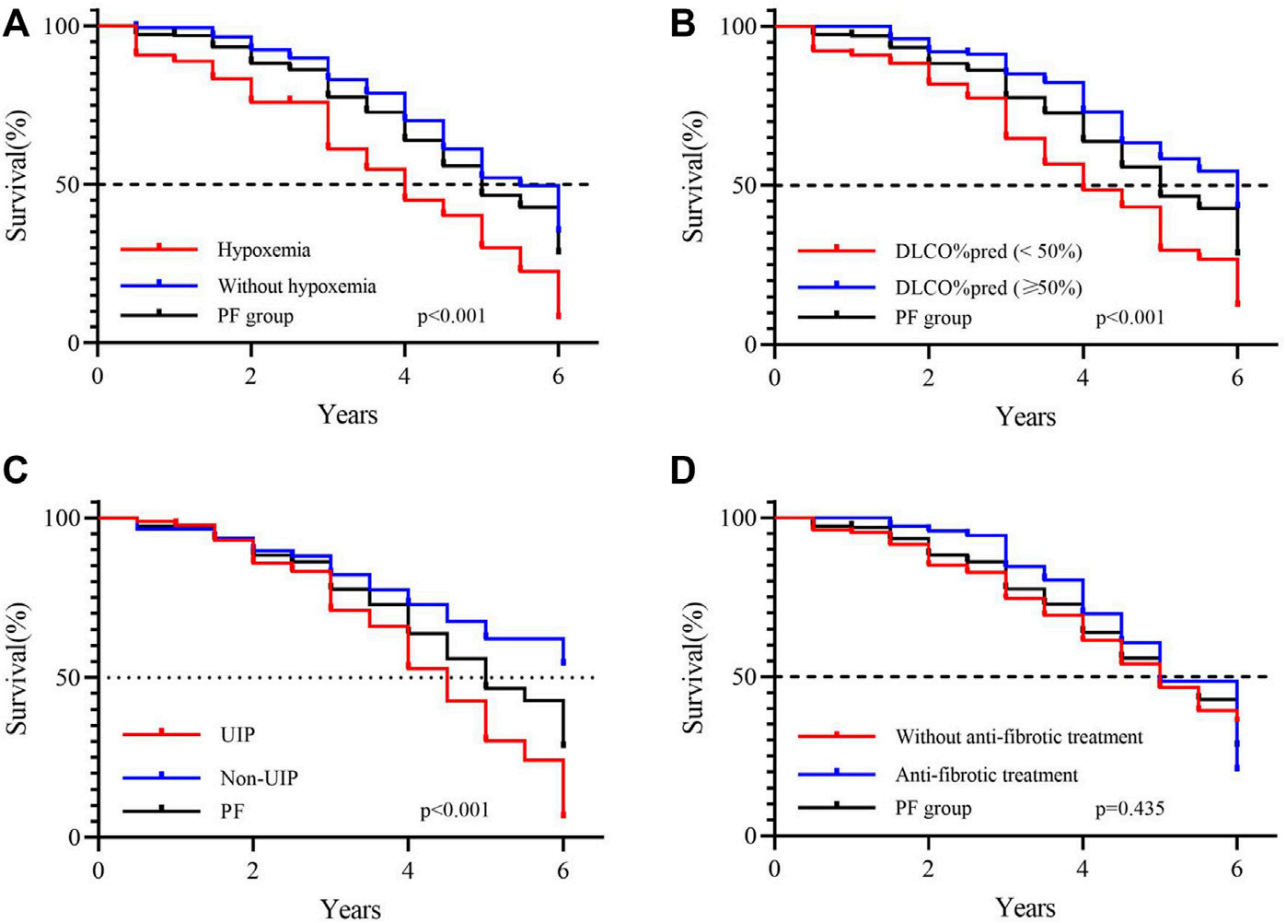
Survival in patients with progressive fibrosing ILD. **(A)** Survival according to with or without hypoxemia at baseline (log-rank test, *p* < 0.001); **(B)** survival according to DLCO% pred at baseline with a 50% threshold (log-rank test, *p* < 0.001); **(C)** survival according to the UIP-like fibrotic pattern on HRCT (log-rank test, *p* < 0.001); **(D)** survival according to with or without anti-fibrotic treatment during observation (log-rank test, *p* = 0.435).

Patients were followed up for a median duration of 3 (2–4) years with 128 (22.1%) deaths during follow-up, and the median overall survival (OS) time for whole patients with fibrosing ILDs was 6 years. In the non-PF group, the median overall survival was not reached, while in the PF-ILD group, 85 of 227 (37.45%) patients died, with a median OS of 5 years (Supplementary Figure S4). The median survival time was shorter in patients with hypoxemia (OS: 4 years), baseline DLCO% predicted <50% (OS: 4 years), and UIP-like pattern (OS: 4.5 years). Subgroup analysis based on anti-fibrotic treatment using the log-rank test showed no significant intergroup difference (*p* = 0.435) (Figure 3). Within patients in the PF group who received anti-fibrotic treatment, IPF patients showed worse OS (OS: 4.5 years) than non-IPF patients (OS: 6 years) (log-rank test, *p* = 0.003, Supplementary Figure S5). Table 4 shows the results of univariate and multivariate Cox analyses of predictors for all-cause mortality. By multivariate Cox analysis, lower DLCO% predicted (HR 2.25, 95% CI 1.45 to 3.50, *p* < 0.001), hypoxemia (HR 2.08, 95% CI 1.31 to 3.32, *p* = 0.002), and UIP-like fibrosis pattern on HRCT (HR 1.68, 95% CI 1.04 to 2.71, *p* = 0.034) were associated with an increased risk of mortality.

**TABLE 4:** Factors associated with 6-year all-cause mortality in the PF-group.

Covariate	Univariable analysis		Multivariable analysis	
	HR (95% CI)	*p*-value	HR (95% CI)	*p*-value
Age	1.57 (0.99–2.47)	0.054	1.1 (0.67–1.82)	0.698
Male	1.06 (0.69–1.64)	0.777	—	—
BMI	1.02 (0.65–1.58)	0.944	—	—
Smoking	1.23 (0.80–1.88)	0.347	—	—
FVC% pred	1.13 (0.66–1.95)	0.66	—	—
DLCO% pred	2.30 (1.50–3.45)	<0.001	2.25 (1.45–3.50)	<0.001
Velcro	0.97 (0.63–1.49)	0.88	—	—
Clubbing of fingers	0.77 (0.49–1.22)	0.269	—	—
Hypoxemia	2.24 (1.43–3.50)	<0.001	2.08 (1.31–3.32)	0.002
Hospitalization at baseline	0.90 (0.50–1.61)	0.719	—	—
UIP-like pattern on HRCT	0.23 (1.45–3.45)	<0.001	1.68 (1.04–2.71)	0.034
WBC	1.19 (0.78–1.83)	0.419	—	—
Neutrophils	1.09 (0.71–1.67)	0.697	—	—
Lymphocytes	1.24 (0.81–1.89)	0.33	—	—
Monocyte	1.19 (0.76–1.82)	0.429	—	—
RDW	1.15 (0.75–1.77)	0.516	—	—
LMR	0.86 (0.56–1.32)	0.482	—	—
NLR	1.16 (0.76–1.78)	0.495	—	—
PLR	0.78 (0.50–1.21)	0.268	—	—
SIRI	1.39 (0.91–2.13)	0.132	1.17 (0.76–1.82)	0.480
AISI	1.08 (0.69–1.67)	0.746	—	—
Anti-fibrotic treatment	0.84 (0.54–1.32)	0.450		

AISI: aggregate index of systemic inflammation; BMI: body mass index; CTD, connective tissue disease; DLCO, diffusion capacity of the lung for carbon monoxide; FVC, forced vital capacity; HRCT, high-resolution computed tomography; MLR, monocyte-to-lymphocyte ratio; NLR, neutrophil-to-lymphocyte ratio; PLR, platelet-to-lymphocyte ratio; RDW: red cell distribution width; SIRI, systemic inflammatory response index; UIP, usual interstitial pneumonia; WBC, white blood cell.

## Discussion

We provided a clinical cohort profile of patients with fibrosing ILD in China. A significant percentage of patients with fibrosing ILD tend to develop progressive fibrosing, which is associated with accelerated decline in lung function secondary to progressive fibrosis and symptomatic worsening (Nasser et al., 2021b). Prevalence of non-IPF PF-ILD varied from 28.57% to 39.07% owing to various diagnosis subgroups in our data, consistent with the findings reported by previous studies (approximately 33.0%) (Flaherty et al., 2019; Nasser et al., 2021b). A large-scale survey estimated that 18.0%–32.0% of patients diagnosed with non-IPF ILDs developed progressive fibrosis with overall survival of 61–80 months in the US, France, Germany, Italy, Spain, the United Kingdom, and Japan (Wijsenbeek et al., 2019). PF-ILD was identified in 135 of 396 (34.1%) patients with non-IPF ILDs in a cohort of Korean patients (Kwon et al., 2021). In another Chinese single-center cohort of 608 patients, 132 patients (21.7%) with ILD met the criteria for PF-ILD (Chen et al., 2021). The difference in findings between previous studies and our study may be attributable to the older age, poorer DLCO% predicted, or various disease classifications used for non-IPF ILDs in our patients. In our study, half of the patients with IPF showed rapid disease progression within 24 months. The clinical course of IPF is variable and difficult to predict, with a median survival time of 2–3 years after diagnosis without anti-fibrotic therapy. However, approximately 25.0% of patients survive over 5 years after the initial diagnosis (Kärkkäinen et al., 2019). Therefore, it is possible that lung function in patients with IPF remained stable or declined only slightly during the first 24 months but eventually led to poor outcomes.

Healthcare utilization is expected to be higher in patients with PF-ILD (Holtze et al., 2018; Wuyts et al., 2020). Hospitalization frequency was higher in the PF-ILD group than in the non-PF-ILD group during follow-up. Patients with PF-ILD received glucocorticoids for primary therapy, which emphasizes the need for alternative treatment. Many clinical trials have confirmed the efficacy of anti-fibrotic drugs to slow disease progression in patients with PF-ILD (Behr et al., 2021; Nambiar et al., 2021). Our data showed a trend of better survival in patients with PF-ILD, though not statistically significant, who received anti-fibrotic treatment; nintedanib and pirfenidone are known to prolong life expectancy in patients with IPF (Costabel et al., 2017; Lancaster et al., 2019). In the INBUILD trial, the annual rate of decline in the FVC in patients with PF-ILD was significantly lower among patients who received nintedanib (−80.8 ml/year) than among those who received a placebo (−187.8 ml/year) (Flaherty et al., 2019). However, only 32.16% of patients receive anti-fibrotic treatment in our real-world clinical settings, which indicates the possible financial burden associated with these drugs.

Overall, the mean lung function measures remained stable during follow-up in the non-PF-ILD group but showed a decline in the PF-ILD group. In the PF-ILD group, the change in the estimated mean annual FVC was −140 ml. In our study, the decline in the FVC in patients with PF-ILD was similar to that reported by the PROGRESS clinical cohort after 52 weeks (−136 ml) (Nasser et al., 2021b). Another study reported that the mean annual FVC change was −69.9 ml in a United States cohort and −50.0 ml in a United Kingdom cohort of patients with PF-ILD (Oldham et al., 2021). These differences may be attributable to the heterogeneous lung function trajectory across the ILD subgroups. Analysis of diagnosis subgroups showed a mean annual FVC change of −37.2 ml in the CTD-ILD, −92.0 ml in the FHP, and −69.5 ml in the IIP groups (Oldham et al., 2021). We did not perform diagnosis stratification owing to the limited sample size. A greater number of studies that perform the stratification are warranted to investigate the lung function trajectory.

We observed that the HRCT-documented UIP-like fibrotic pattern in patients with fibrosing ILDs was associated with more rapid disease progression, which suggests that morphological patterns may be prognostically significant; our findings are consistent with those reported by previous studies (Nasser et al., 2021b). Moreover, our study showed that clubbing of fingers (enlargement of the ends of the finger accompanied by a downward sloping of the nails) significantly predicted PF-ILD. A previous study reported that the prevalence of clubbing of fingers in cases of fibrosing ILDs ranged from 7.0% to 42.0% (van Manen et al., 2017). Clubbing of fingers is considered an essential clinical finding in patients with fibrosing ILDs and represents decreased oxygen levels and poor prognosis (Kanematsu et al., 1994; van Manen et al., 2017).

Hypoxemia, low DLCO% predicted, and HRCT-documented UIP pattern were associated with worse mortality in patients with PF. Reportedly, male sex, older age, lower FVC or DLCO at baseline, decline in lung function compared with the baseline, subgroups based on diagnosis, HRCT-documented UIP-like fibrotic pattern, or honeycombing were indicators of poor prognosis (Wongkarnjana et al., 2020). Cluster analysis was used to classify patients with ILD into clinical subgroups based on their clinical courses. Patients with the most rapid decline in lung function or fibrosis progression and poor survival were usually clustered together in ILDs or IP with myositis-specific autoantibodies (Adegunsoye et al., 2018; Lia et al., 2022). These findings suggest that evidence of progressive pulmonary fibrosis may be useful to define a group of patients with fibrosing ILD of multifactorial etiologies and poor prognoses. Although accurate and prompt diagnosis is important to enable initiation of optimal management, it is possible to identify patients at high risk of progression through observation of disease behavior despite the specific diagnosis. Validation cohorts are required to confirm mortality predictors.

Given the possible relationship between prognosis and biomarkers obtained by the routinely provided blood cell count test in IPF, we sought to explore the prognostic capacity of the blood cell count and the combined indexes in PF-ILDs. A pooled retrospective analysis of 2,067 IPF patients derived from the clinical trials showed that patients with higher monocyte count had a higher 1-year risk of progression, all-cause mortality, and all-cause hospitalization (Kreuter et al., 2021). On the contrary, we did not observe the relationship between disease progression or mortality and blood cell count and the combined index. According to a multicenter retrospective study, higher baseline NLR and absolute monocyte counts predict worse survival in IPF but not in fHP (Barratt et al., 2021), highlighting the potential divergence in the underlying mechanisms of these diseases. However, our sample size is smaller than the previous reports for the prognostic role of blood cell counts. Second, single peripheral blood cell measures may be influenced by many short-term factors. Further multicenter studies with a larger population would be informative about the applicability of blood cell counts in predicting prognosis.

We provided data on longitudinal clinical outcomes in patients with fibrosing ILD from China. Patients evaluated in a tertiary referral center through a multidisciplinary discussion were routinely followed up every 6–12 months with pulmonary function tests, HRCT, and visits. Unlike previous studies for FP-ILDs (Flaherty et al., 2019; Wijsenbeek et al., 2019; Nasser et al., 2021b) which generally emphasized non-IPF patients, our cohort included both IPF and non-IPF patients as a whole group and also provided separate data for both groups. In this way, the exploration between clinical characteristics and prognosis of patients with PF-ILD is of more clinically utility because it is hard to make specific diagnosis for some ILDs at an early stage, especially for IPF. In addition, we tried to study the relationship between the patient’s blood count values and prognosis, which was not explored in PF-ILD patients as a whole group before. The negative results were supported by some previous research studies while opposed by others studying specific diagnosis group of diseases, highlighting cautious use of routine blood test values as prognosis predictors.

Several limitations should be considered: 1) the retrospective and monocentric design of this study may have led to a selection bias. 2) This was a small-scale study in which we observed a reduction in available data on lung function over time, which resulted in an inaccurate estimation of the lung function trajectory. 3) Acute exacerbations, as one of the leading causes of death in patients with IPF (annual incidence of up to 20.0%) (Richeldi, 2015; Kolb et al., 2018; Ruaro et al., 2021; Yoon et al., 2021), were not investigated because some exacerbations were not recorded in the medical system of our hospital. The annual incidence of acute exacerbations in patients with non-IPF PF-ILD was 13.9% (Nasser et al., 2021b; Petnak et al., 2021; Yoon et al., 2021). Further studies are warranted to thoroughly investigate the effects of acute exacerbations. 4) We used all-cause mortality rather than ILD-related mortality as the outcome because all deaths did not occur at the hospital, and some causes of death were not reported reliably.

In summary, we presented a hospital population-based cohort profile of patients with PF-ILDs in China. We reported that 28.57%–53.10% of patients with fibrosing ILD developed a progressive fibrosing feature. Patients with clubbing of fingers or an HRCT-documented UIP pattern showed a high potential risk for PF-ILD. The risk factors associated with mortality in patients with PF-ILD included hypoxemia, low DLCO% predicted, or an HRCT-documented UIP pattern. The relationship between prognosis and blood cell count and the combined index was not observed, highlighting the divergence in the underlying mechanisms of PF-ILDs.

## Data Availability

The original contributions presented in the study are included in the article/Supplementary Material; further inquiries can be directed to the corresponding author.

## Abbreviations

AISI, aggregate index of systemic inflammation; BMI, body mass index; CI, confidence interval; CTD, connective tissue disease; DLCO, diffusion capacity of the lung for carbon monoxide; FHP, fibrotic hypersensitivity pneumonitis; FVC, forced vital capacity; HR, hazard ratio; HRCT, high-resolution computed tomography; iNSIP, idiopathic non-specific interstitial pneumonia; IIP, idiopathic interstitial pneumonia; ILD, interstitial lung disease; IPF, idiopathic pulmonary fibrosis; IQR, interquartile range; MLR, monocyte-to-lymphocyte ratio; NLR, neutrophil-to-lymphocyte ratio; OR, hazard ratio; PF, progressive fibrosing; PFT, pulmonary function testing; PLR, platelet-to-lymphocyte ratio; pSS-ILD, primary Sjögren syndrome-associated ILD; SD, standard deviation; RDW: red cell distribution width; SIRI, systemic inflammatory response index; SSc-ILD, sclerosis-associated ILD; UIP, usual interstitial pneumonia; WBC, white blood cell.
