# Supplementary material for: Prognostic Predictive Characteristics in Patients With Fibrosing Interstitial Lung Disease: A Retrospective Cohort Study
Source: Front Pharmacol. 2022 Jul 1;13:924754. doi: 10.3389/fphar.2022.924754 (PMC9283576; doi:10.3389/fphar.2022.924754)
Supplement: Supplementary file 1 [file Table1.DOCX]

**Supplementary Material**

**Contents**

**Figure S1- Venn Diagram for patients meeting the criteria for PF-ILD 2**

**Figure S2- Comorbidity information for PF-ILD and non-PF ILD 3**

**Figure S3- Percentages of the patients developing PF-ILD 4**

**Figure S4- Overall survival 5**

**Figure S5- Survival within the PF group who received antifibrotic treatment stratified by diagnosis 6**

**Table S1- Treatments received during the follow-up 7**

**Table S2- Factors associated with 6 years all-cause mortality 8**

**
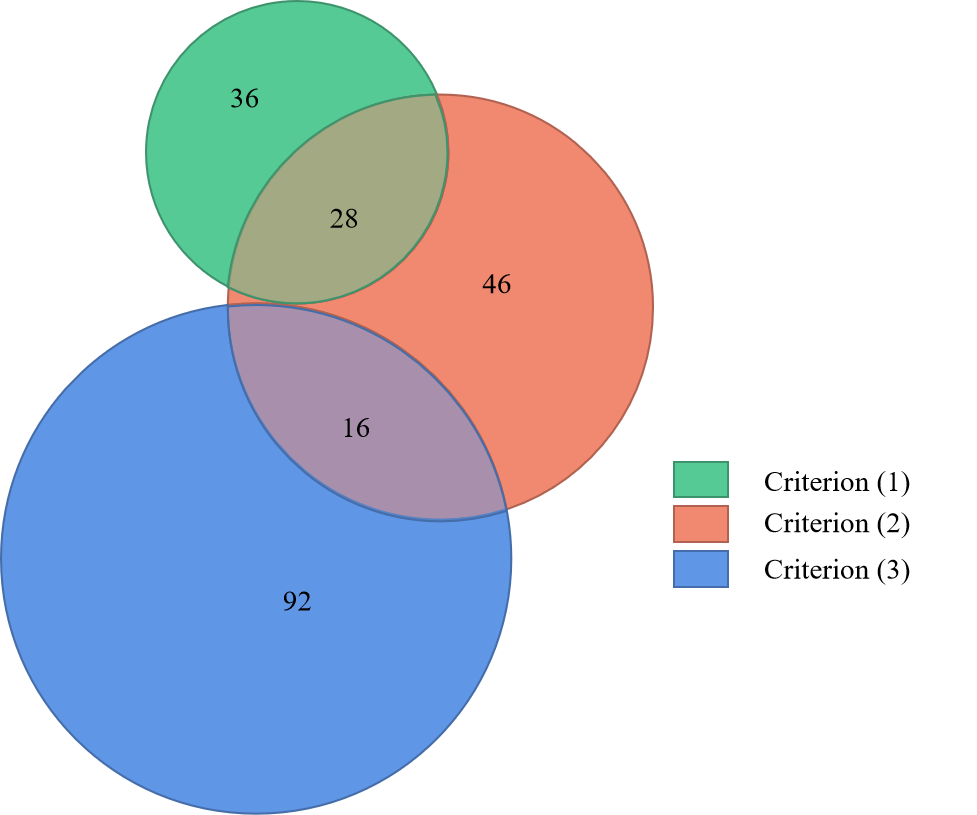
**

**Figure S1. Venn Diagram for patients meeting the criteria for PF-ILD.**

Criterion (1): a relative decline of ⩾10% in FVC; Criterion (2): relative decline of ⩾15% in diffusion capacity of the lung for carbon monoxide (DLCO); Criterion (3): worsening symptoms and/or worsening radiological findings accompanied by ⩾5 to <10% relative decrease in FVC.

**
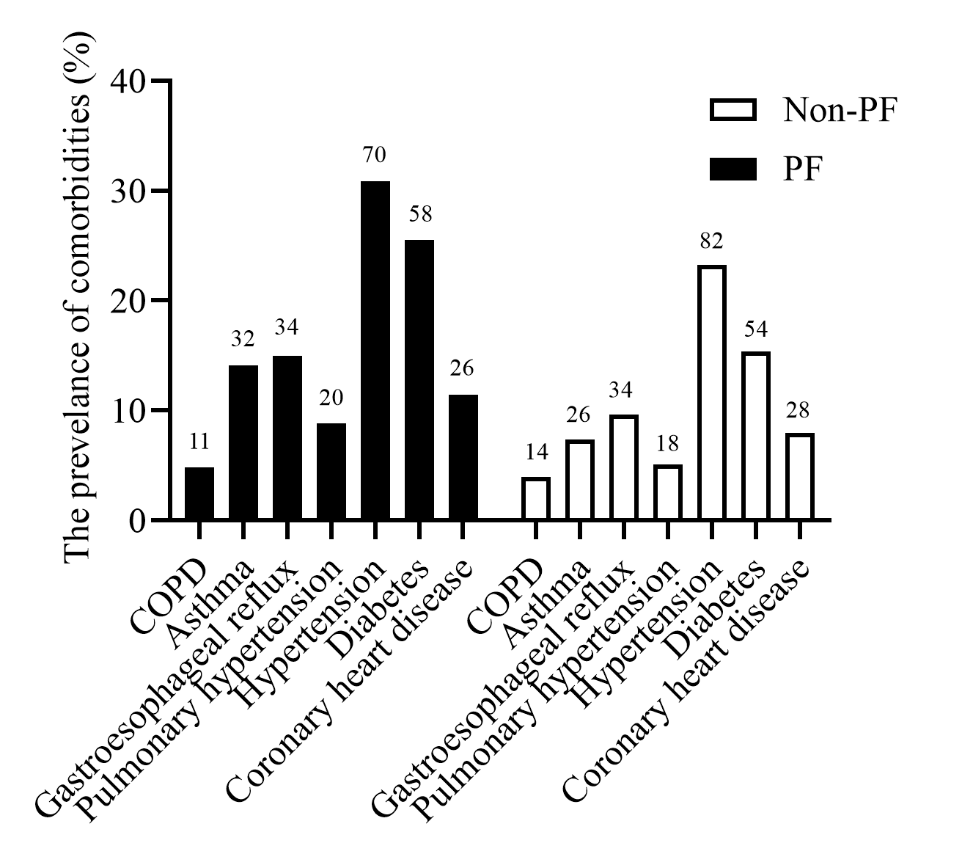
**

**Figure S2. Comorbidity information for PF-ILD and non-PF ILD.** The prevalence for COPD, asthma, gastroesophageal reflux disease, pulmonary hypertension, hypertension, diabetes and coronary heart disease was 4.85%, 14.10%, 14.98%, 8.81%, 30.84%, 25.55%, 11.45% in PF group (n=227) and 3.98%, 7.39%, 9.66%, 5.11%, 23.30%, 15.34%, 7.95% in non-PF group (n=352), respectively.

**^#^**Only 5 patients were considered with pulmonary hypertension assessed by right heart catheterization. Others were based on echocardiography (estimated pulmonary artery pressure ≥ 30mmHg).

Abbreviations: COPD: chronic obstructive pulmonary disease.

**
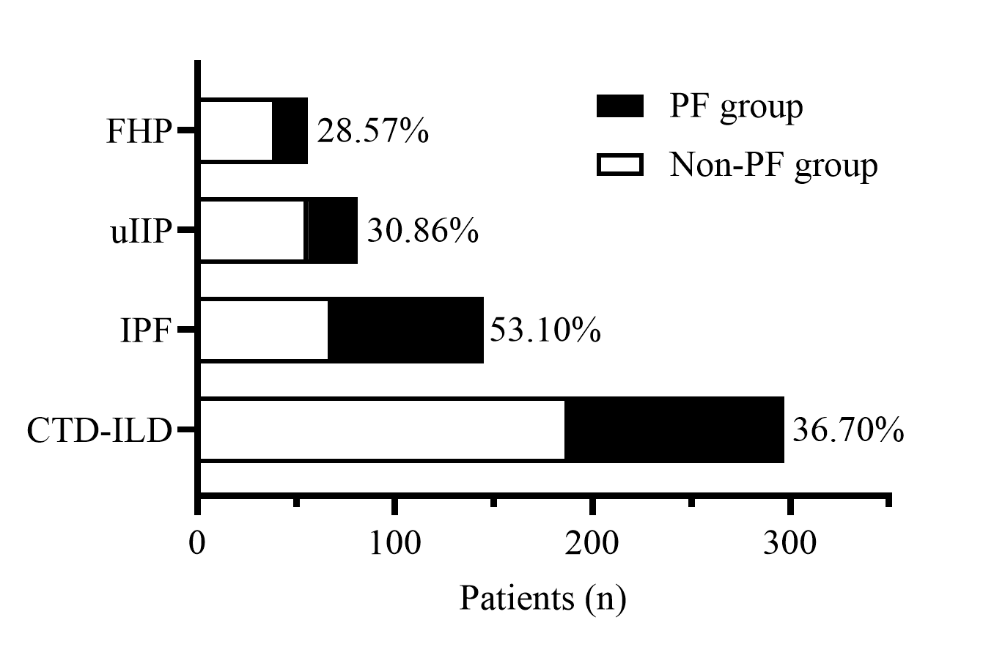
**

**Figure S3. Percentages of the patients developing PF-ILD.**

Abbreviations: PF: progressive fibrosis, CTD-ILD: connective tissue disease-associated interstitial lung disease, FHP: fibrotic hypersensitivity pneumonitis, uIIP: unclassifiable idiopathic interstitial pneumonia, IPF: idiopathic pulmonary fibrosis.


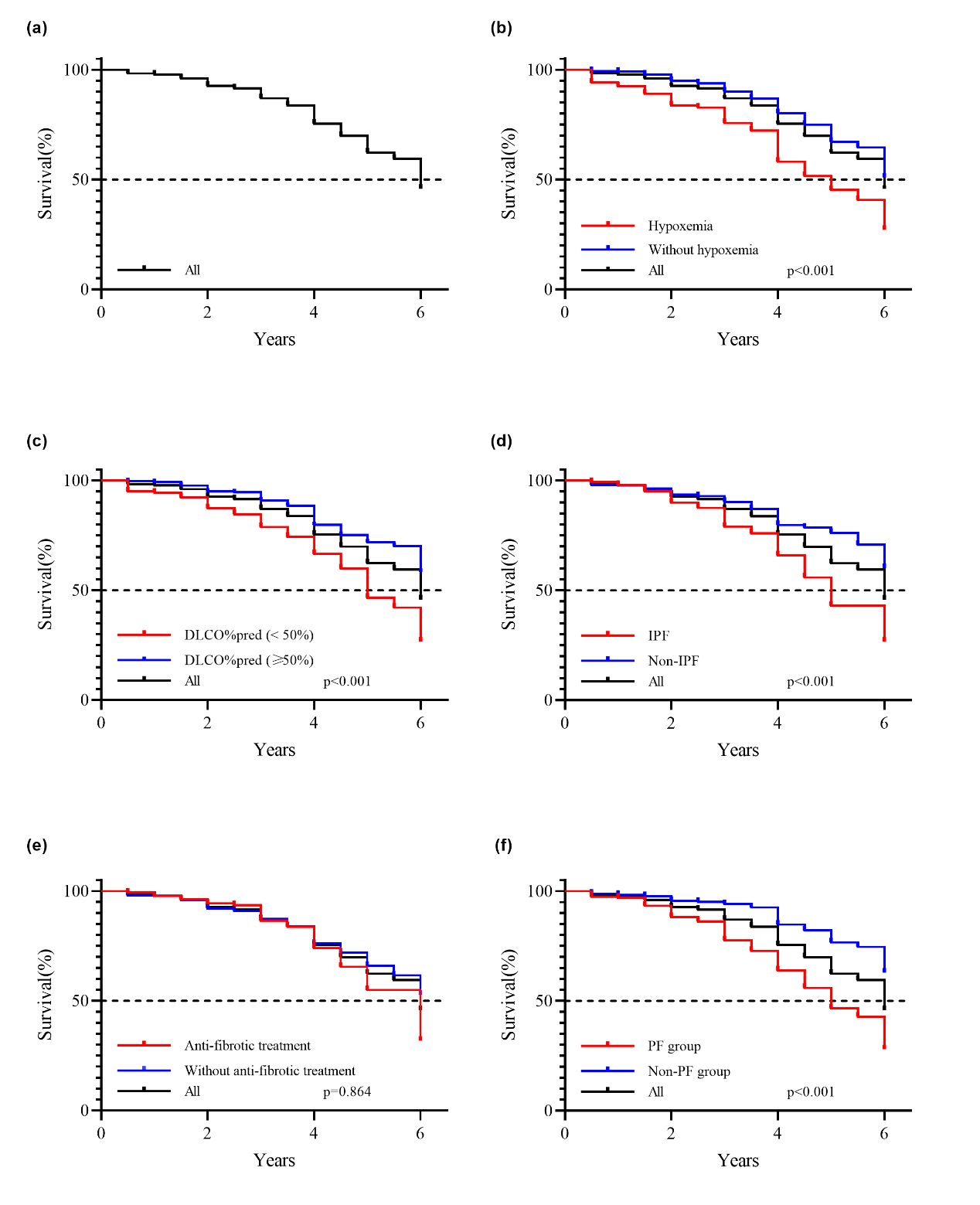


**Figure S4. Overall survival.** (a) Overall patient cohort; (b) overall survival according to with or without hypoxemia at baseline (Log-rank test, p<0.001); (c) overall survival according to DLCO% pred at baseline with a 50% threshold (Log-rank test, p<0.001); (d) overall survival according to IPF diagnosis (Log-rank test, p<0.001); (e) overall survival according to with or without anti-fibrotic treatment during observation (Log-rank test, p=0.864); (f) overall survival according to with or without progressive fibrosing feature during observation (Log-rank test, p<0.001).


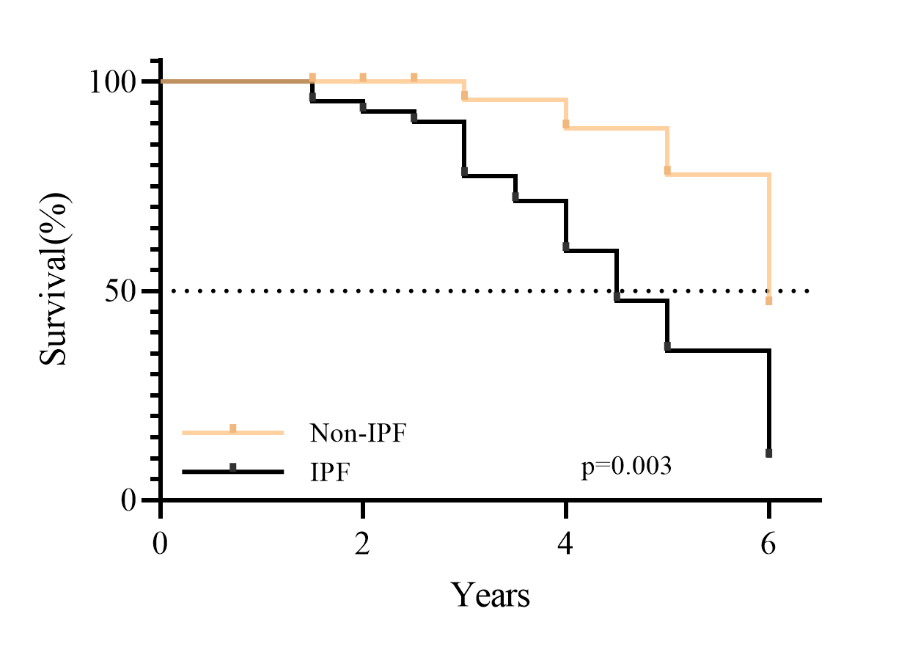


**Figure S5.** **Survival within the PF group who received antifibrotic treatment stratified by diagnosis.** (Log-rank test, p=0.003)

**Table S1. Treatments received by the patients in a cohort at any time during the course of the study period**

| Treatments | | All (n=579) | PF-ILD group (n=227) | Non-PF-ILD group (n=352) | *P*-value |
| --- | --- | --- | --- | --- | --- |
|  | Glucocorticoids | 364 | 133(58.59) | 231(65.63) | 0.087 |
|  | Immunosuppressive agents | 218 | 83(36.56) | 135(38.35) | 0.665 |
|  | Anti-fibrotic treatment | 130 | 73(32.16) | 57(16.19) | <0.001 |
|  | Oxygen therapy | 59 | 28(12.33) | 31(8.81) | 0.171 |

Values were given as n (%). Abbreviations: PF-ILD: progressive fibrosing interstitial lung disease.

**Table S2. Factors associated with 6 years all-cause mortality in fibrosing ILDs**

| Covariates | Univariable analysis | | Multivariable analysis | |
| --- | --- | --- | --- | --- |
|  | OR (95% CI) | *P* value | OR (95% CI) | *P* value |
| Age | 1.75（1.20-2.55） | 0.004 | 1.46 (0.98-2.17) | 0.061 |
| Male | 1.38 (0.96-1.97) | 0.083 | 1.33 (0.91-1.74) | 0.147 |
| BMI | 0.90 (0.63-1.29) | 0.550 | - | - |
| Smoking | 1.26 (0.89-1.78) | 0.200 | 0.99 (0.68-1.45) | 0.973 |
| FVC% pred | 1.14 (0.75-1.72) | 0.548 | - | - |
| DLCO% pred | 2.25 (1.60-3.18) | <0.001 | 2.35 (1.64-3.36) | <0.001 |
| Velcro | 1.25 (0.86-1.72) | 0.272 | - | - |
| Clubbing of fingers | 0.94 (0.64-1.37) | 0.726 | - | - |
| Hypoxemia | 2.29 (1.59-3.26) | <0.001 | 1.59 (1.05-2.39) | 0.028 |
| Hospitalization at baseline | 1.46 (0.98-2.16) | 0.06 | 2.10 (1.45-3.06) | <0.001 |
| UIP-like pattern on HRCT | 2.36 (1.66-3.34) | <0.001 | 1.75 (1.18-2.59) | 0.005 |
| WBC | 1.12 (0.79-1.58) | 0.529 | - | - |
| Neutrophils | 1.12 (0.79-1.59) | 0.521 | - | - |
| Lymphocytes | 0.91 (0.64-1.29) | 0.598 | - | - |
| Monocyte | 1.14 (0.81-1.62) | 0.455 | - | - |
| RDW | 1.25 (0.88-1.7) | 0.207 | - | - |
| LMR | 0.99 (0.70-1.41) | 0.954 | - | - |
| NLR | 1.10 (0.78-1.56) | 0.588 | - | - |
| PLR | 0.79 (0.56-1.12) | 0.183 | - | - |
| SIRI | 1.23 (0.87-1.75) | 0.242 | - | - |
| AISI | 1.03 (0.73-1.46) | 0.852 | - | - |
| PF | 2.84 (0.89-1.87) | <0.001 | 2.61 (1.78-3.83) | <0.001 |
| Anti-fibrotic treatment | 1.29 (1.54-3.10) | 0.187 | 0.65 (0.43-0.97) | 0.036 |

Abbreviations: AISI: aggregate index of systemic inflammation; BMI: body-mass index; CTD: connective tissue disease; DLCO: diffusion capacity of the lung for carbon monoxide; FVC: forced vital capacity; HRCT: high-resolution computed tomography; MLR: monocyte-to-lymphocyte ratio; NLR: neutrophil-to-lymphocyte ratio; PF: progressive fibrosis; PLR: platelet-to-lymphocyte ratio; SII: systemic inflammatory index; SIRI: systemic inflammatory response index; UIP: usual interstitial pneumonia; WBC: white blood cell.
